# Timed Sequence Task: A New Paradigm to Study Motor Learning and Flexibility in Mice

**DOI:** 10.1523/ENEURO.0145-23.2023

**Published:** 2023-10-03

**Authors:** Anna Urushadze, Milan Janicek, Alice Abbondanza, Helena Janickova

**Affiliations:** 1 Laboratory of Neurochemistry, Institute of Physiology of the Czech Academy of Sciences, 14200 Prague, Czech Republic; 2 Central Library, Charles University, 11636 Prague, Czech Republic; 3 CNRS UMR 8246, INSERM U1130, Neuroscience Paris Seine-Institut de Biologie Paris Seine (NPS-IBPS), Sorbonne Université, 75005 Paris, France

**Keywords:** flexibility, mice, motor learning, operant learning

## Abstract

Motor learning and flexibility allow animals to perform routine actions efficiently while keeping them flexible. A number of paradigms are used to test cognitive flexibility, but not many of them focus specifically on the learning of complex motor sequences and their flexibility. While many tests use operant or touchscreen boxes that offer high throughput and reproducibility, the motor actions themselves are mostly simple presses of a designated lever. To focus more on motor actions during the operant task and to probe the flexibility of these well trained actions, we developed a new operant paradigm for mice, the “timed sequence task.” The task requires mice to learn a sequence of lever presses that have to be emitted in precisely defined time limits. After training, the required pressing sequence and/or timing of individual presses is modified to test the ability of mice to alter their previously trained motor actions. We provide a code for the new protocol that can be used and adapted to common types of operant boxes. In addition, we provide a set of scripts that allow automatic extraction and analysis of numerous parameters recorded during each session. We demonstrate that the analysis of multiple performance parameters is necessary for detailed insight into the behavior of animals during the task. We validate our paradigm in an experiment using the valproate model of autism as a model of cognitive inflexibility. We show that the valproate mice show superior performance at specific stages of the task, paradoxically because of their propensity to more stereotypic behavior.

## Visual Abstract

**Figure:**
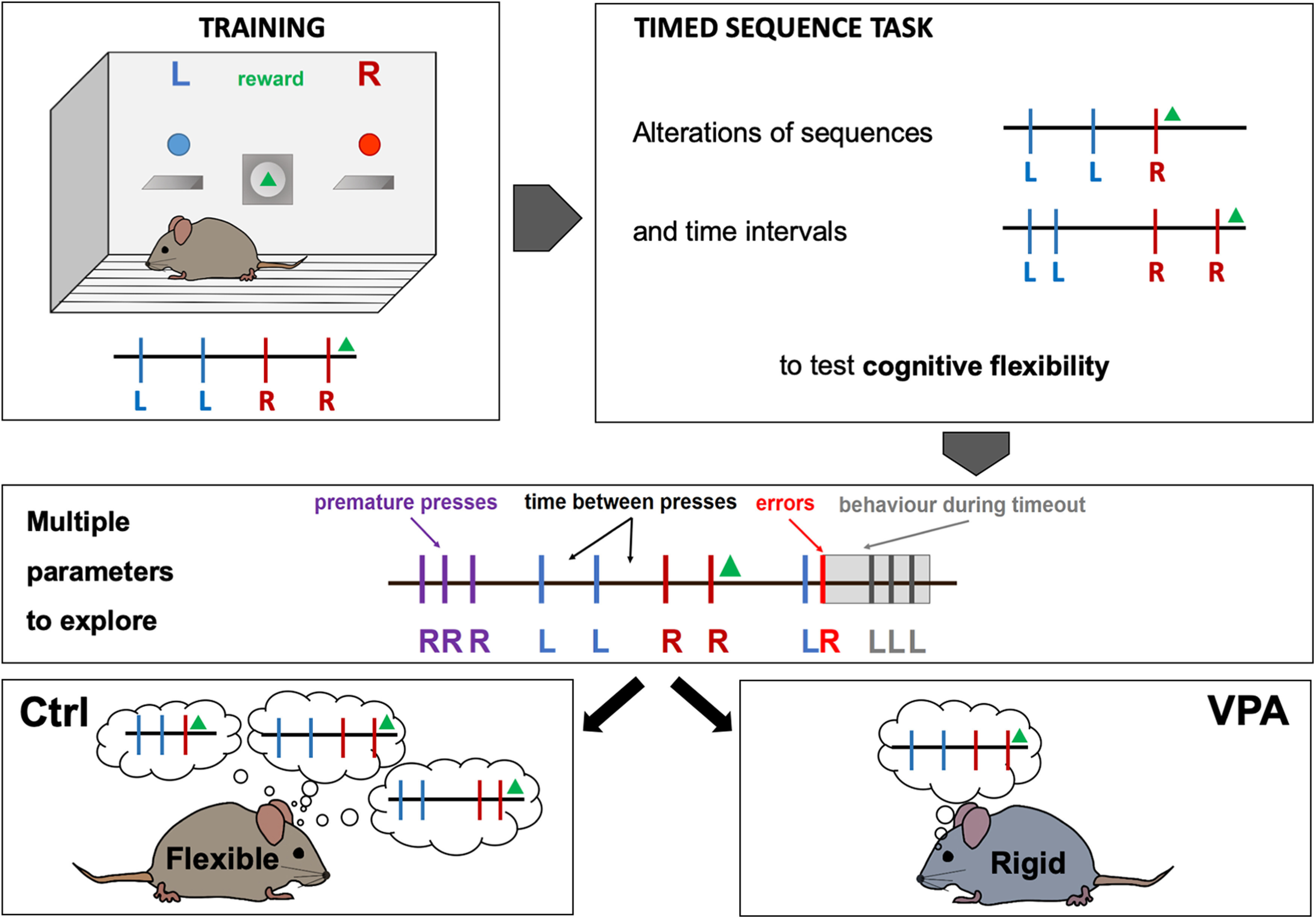

## Significance Statement

Cognitive flexibility impairment is a crucial component of many neurologic disorders, and it is frequently evaluated in animal models. As the commonly used tests usually do not focus on motor learning and the ability to adapt motor sequences, we designed a new paradigm to evaluate motor learning and its flexibility. The timed sequence task is automatized and easily accessible as it is based on widely available operant boxes. During the training, the task requires precise timing of each action to force stereotypic performance. Its relative complexity allows detailed analysis of multiple parameters and therefore detailed insight into the behavior of an animal. The task can be used to reveal and understand subtle differences in motor and operant learning and flexibility.

## Introduction

Acquisition of new motor actions and their successful automatization is a crucial part of the behavior of animals. The animals have to be able to not only learn the motor action but also flexibly change it when required. Various tasks for testing of cognitive flexibility are performed in conventional operant or in touchscreen-based boxes (Mar et al., 2013). Alternatively, a variety of maze-based tasks (Vorhees and Williams, 2006) can be used. In most of these paradigms, the original motor action is relatively simple (a lever press or a nose poke) and in the flexibility phase, it is transformed into its mirror image. In these paradigms, it may be difficult to capture subtle changes in the behavior of an animal as a simple motor action does not allow for a detailed insight (Keeler et al., 2014). In the literature, several paradigms can be found that are suitable for testing more complex motor learning and automatization (Keeler et al., 2014; Geddes et al., 2018; Dhawale et al., 2021; Janickova et al., 2021; Turner et al., 2022; Wolff et al., 2022). However, these tasks usually do not involve flexibility, and, more importantly, they often require specialized equipment and/or technical expertise, which makes them less accessible. Notably, some of these more complex tasks have so far been performed only with rats, and their adaptability to mice may not prove easy.

To overcome the aforementioned difficulties, we created a timed sequence task where mice perform a heterogeneous sequence of four presses in a highly stereotypic way. To ensure this, mice are taught to perform the individual presses within predefined time limits, to make the actions in individual trials highly uniform. Once the complex sequence is well trained, it can be repeatedly modified at different steps, to implement the flexibility component of the task. In addition, the task structure allows collection of data and analysis of numerous behavioral parameters. The present protocol can be realized with commonly available operant boxes equipped only with two nonretractable levers and a feeder. The protocol code is programmed in a proprietary but common programming language that is used with a common type of operant boxes. The code is open and can be easily modified to create new variations of the task. To shorten the tedious analysis of multiple task parameters, we also provide a set of scripts in Python for automatized processing of the data and for user-friendly manual checking of lengthy task logs if needed. As a first choice, we tested our new protocol in the valproate mouse model of autism (Nicolini and Fahnestock, 2018), a model with a prominent social impairment, impairment of motor skills, and a higher propensity to habitual and repetitive behavior (Nicolini and Fahnestock, 2018; Tartaglione et al., 2019). In this model, we show specific alterations in several parameters of the task, together providing evidence of increased habitual performance and slight motor impairment in these animals.

## Materials and Methods

### Animals and preparation of the valproic acid model

Male mice of C57BL/6J genetic background were used and purchased from Charles River Laboratories. They were group housed in a room with controlled temperature and 12 h light/dark cycle. All experimental procedures complied with the directive of the European Community Council on the use of laboratory animals (2010/63/EU) and were approved by the animal care committee of the Czech Academy of Sciences. The behavioral testing started at 2 months of age. Prenatal exposure to valproic acid (VPA) was used to induce autistic phenotype, according to the published protocol (Lucchina and Depino, 2014). Pregnant females were injected subcutaneously at gestation day 12.5 with VPA sodium salt (600 mg/kg; Sigma-Aldrich) in a concentration of 5 ml/kg or, for controls, with vehicle only (saline). In total, we produced 11 control males and 11 males exposed to VPA.

### Confirmation of the autistic phenotype

In 2-month-old mice, we confirmed the autistic phenotype (Ergaz et al., 2016) using three tests in the following order: social preference test (Nadler et al., 2004), hole-board test to examine compulsivity (Martos et al., 2017), and sticky tape test to evaluate dexterity (Bouet et al., 2009). The social preference test was performed in a three-chamber apparatus (90 × 23 × 23 cm). A mouse was placed in the central chamber and habituated for 5 min. A wire mesh pen cup was allocated in each lateral chamber, with one side containing a juvenile male. During 10 min test session, the time spent by interacting with the juvenile or with the empty cup was manually scored. For the hole-board test (Martos et al., 2017), mouse was placed in a box (40 × 40 cm) equipped with a double floor containing 16 equidistant holes. We recorded sequences of nose pokes into individual holes during 30 min and calculated the probability of returning to the same hole. For the sticky tape test (Bouet et al., 2009), triangle-shaped pieces of tape were attached to the front limbs and the time until the removal of both tapes was measured, with a maximum time of 5 min. The procedure was repeated 3× for each mouse, and averages from the two shortest times were analyzed.

### Timed sequence task

#### Overview

**Figure 1.**
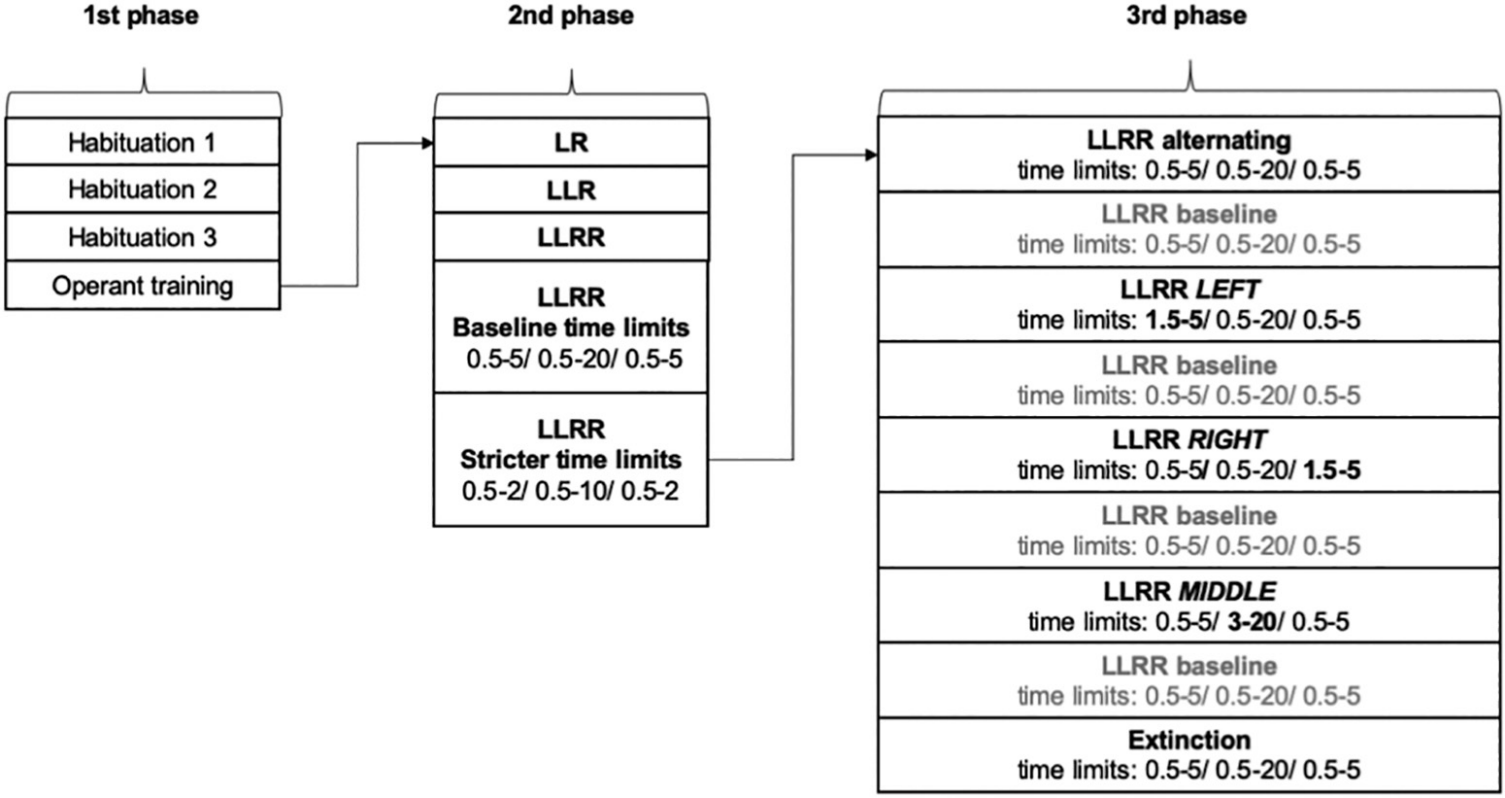
Schematic of the training stages during the timed sequence task. The training consists of the following 3 main phases: (1) basic operant training, (2) acquisition of the timed LLRR sequence, and (3) alterations of the previously learned timed sequence. In the last stage, each alteration is followed by 1–3 sessions of the baseline timed sequence protocol to ensure that mice return to their baseline performance.

The task can be performed in standard operant boxes equipped with fixed levers. In the timed sequence task, mice have to learn a precisely timed sequence of lever presses without any error or interruption. The sequence is acquired in several stages. After successful acquisition, the sequence or the time intervals can be changed in multiple probe sessions to examine the ability to change the previously learned motor program. Each set of probe sessions is followed by baseline sessions to re-establish the original performance. To perform the task, we used a standard operant box (catalog #MED-307A-B1, Med Associates) placed in a sound-attenuating cubicle and operated by Med-PC V software (Med Associates). The box featured two fixed levers and a feeder located between them for the automatic delivery of reward pellets. Above each lever, a cue light was located indicating when the lever was active and should be pressed. The correct execution of each trial was rewarded with a chocolate cereal pellet (20 mg; Bio-Serv). The main house light in the box was kept off throughout the training. Mice underwent daily training sessions between 8:00 A.M. and 5:00 P.M., five times per week. The completion of the whole training and testing required ∼40 training/testing days (8 weeks) for a control mouse. The majority of animals were able to learn the task and complete the whole procedure. However, we defined criteria in Table 1 for each stage to remove nonperforming animals from the study. The individual stages of the training and testing are listed in Figure 1 and Table 1, and in details described below. The respective codes for each stage are available at https://doi.org/10.5281/zenodo.7881104.

**Table 1:** Exclusion criteria used for the individual training stages of the task

Training stage	Exclusion criteria
Habituation 1	No exclusion criteria
Habituation 2 Habituation 3	Mouse does not reach performance criteria within 10 sessions
Operant training	
LR sequence LLR sequence LLRR sequence	Mouse does not reach performance criteria within 10 sessions
LLRR with baseline time limits LLRR with stricter time limits	Mouse does not reach performance criteria within 10 sessions and performs <10 trials on the last (10th) session
LLRR alternating: 3× LLRR + 1× LLR	No exclusion criteria
LLRR baseline	Mouse performs <10 trials on the last (third) baseline session
LLRR *LEFT*	No exclusion criteria
LLRR baseline	Mouse performs <10 trials on the last (third) baseline session
LLRR *RIGHT*	No exclusion criteria
LLRR baseline	Mouse performs <10 trials on the last (third) baseline session
LLRR *MIDDLE*	No exclusion criteria
LLRR baseline	Mouse performs <10 trials on the last (third) baseline session
Extinction	No exclusion criteria

### Food restriction

For the food-motivated testing, 2 weeks before the training initiation, mice were weighed and the range of 85–90% of their original weight was determined as the target weight. Mice were weighed and fed daily to maintain their target weight throughout the training and testing.

### Timed sequence task: description of individual stages

#### First phase, operant training

##### Habituation 1

On the first day of training, mice were placed in the operant box with the feeder off for 10 min. No reward was placed in the feeder, and all mice automatically advanced to the next training step on the next day.

##### Habituation 2

At the beginning of this session, four reward pellets were manually placed into the feeder, and mice were left in the box for 10 min. At the end of the session, the pellets were checked and only the mice that consumed all pellets advanced to the next training stage. Otherwise, the session was repeated.

##### Habituation 3

During the last habituation, mice stayed in the box for 30 min while the reward was automatically delivered every 2 min. Mice had to consume all 15 reward pellets to advance to the next stage. Otherwise, the session was repeated.

##### Operant training

After habituation, mice had to associate lever presses with the delivery of the reinforcement. During the initial operant training, every [left (L) and right (R)] lever press was rewarded, and the levers became active immediately after the reward delivery. The session ended after mice earned 40 rewards or after 30 min, whichever occurred first. After mice successfully earned 40 rewards within a session, they were moved to the next stage. Otherwise, the session was repeated.

#### Second phase, LLRR acquisition

##### LR sequence

To avoid any side bias, mice were initially trained to perform an LR sequence. To obtain a reward, mice had to complete the whole LR sequence with no errors (i.e., sequences “LLR” or “RLR” were not rewarded). After every error, a 5 s time-out (TO) interval was introduced during which the presses were not counted as correct and did not yield any reward. The session ended after 60 min or when the mouse earned 40 rewards, whichever occurred first. To proceed to the next training stage, mice had to earn 40 rewards within the session per day for 3 consecutive days. This criterion and TO settings remained the same for all stages of the second phase. The 40 rewards criterion was selected based on our pilot studies as it was feasible and yet sufficiently challenging for mice.

##### LLR sequence

This stage was similar to the previous with the addition of another left press, forming the sequence LLR (left, left, right).

##### LLRR sequence

The final sequence LLRR (left, left, right, right) consisted of four presses that had to be performed without any errors to be rewarded. Up to this point, intervals between the correct presses were not specified.

##### LLRR with baseline time limits

**Figure 2.**
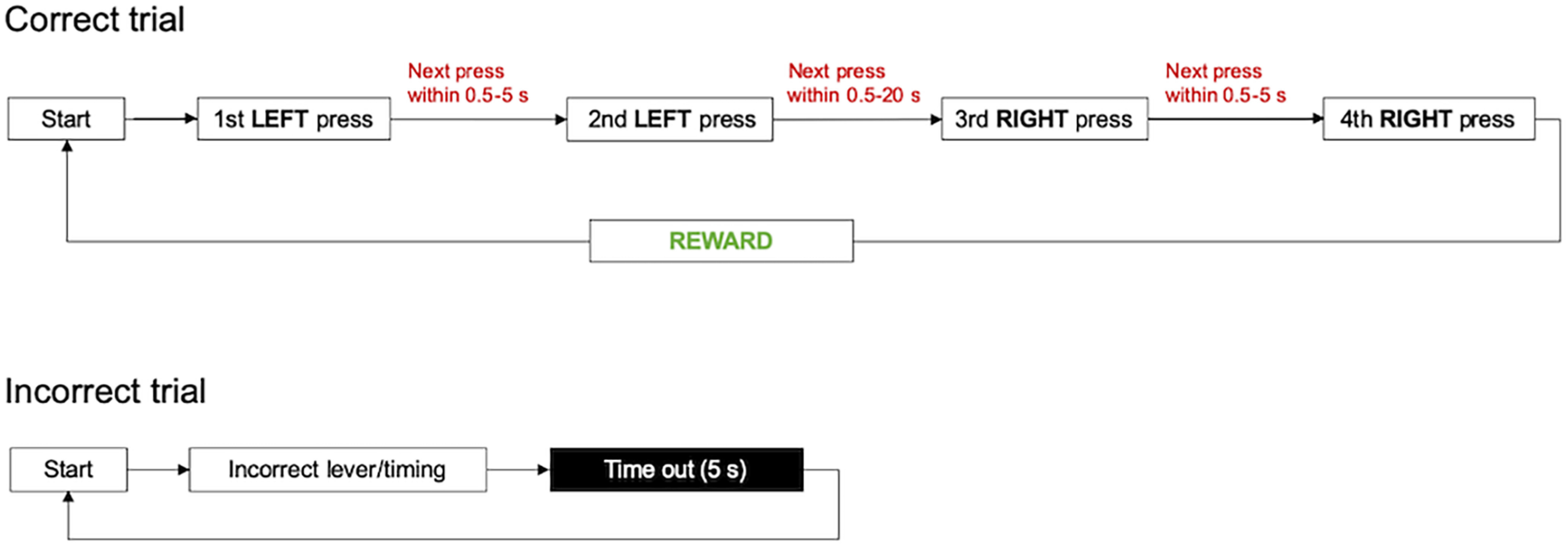
Illustration of the correct and incorrect trial in the baseline timed LLRR sequence. Only complete correct sequences performed within the predefined time intervals are rewarded. After each incorrect trial (resulting from an incorrect press or a timing error), a 5 s time-out is introduced.

At this stage, every press had to be emitted within a certain interval after the preceding press. We call the time limits introduced at this stage as “baseline time limits,” and this particular timed sequence was used for the re-establishing of the baseline performance throughout the following testing. The example illustration of a correct and incorrect trial at this stage is shown in Figure 2. The time intervals we used were 0.5–5 s between the first and second press (interval L–L), 0.5–20 s between the second and third press (L–R), and again 0.5–5 s between the third and fourth press (R–R). To obtain the reward, the sequence had to be performed with no errors and interruptions, within the predefined time limits. The cue lights indicated the activation of the respective lever after the previous press. As in the preceding training stages, the punishment 5 s TO was introduced after either every pressing or every timing error.

##### LLRR with stricter time limits

At this stage, the mice had to perform the sequence within stricter time limits while all other parameters remained the same. The limits were set as 0.5–2 s (L-L), 0.5–10 s (L-R), and 0.5-2 s (R-R). At this stage, some mice started having difficulties to fulfill the criterion. If this was the case, we trained the animals for the maximum of ten d and after that, we moved them automatically to the next stage (Table 1, exclusion criteria).

#### Third phase, LLRR alterations

##### LLRR alternating: 3× LLRR + 1× LLR

After training mice in the baseline timed sequence, we started alternating the pressing sequence and its timing to examine the cognitive flexibility of the animals. First, we tested the effect of an alternated sequence by changing the fourth sequence during the session to LLR. Therefore, in every fourth trial the last press had to be omitted and potential execution of the second right press was counted as an error. Hence, the mouse could choose either the earlier reward or the stereotypical completion of the sequence before getting the pellet. The time intervals between the presses were the same as in the baseline LLRR sequence. No performance criteria were set from this stage onward, and all mice were tested in the alternated sequence for 3 consecutive days. After that, they were run in one to three baseline sessions (LLRR sequence with baseline limits) until they earned 40 rewards at baseline or for the maximum of 3 d.

##### LLRR LEFT

After re-establishing the baseline performance, we started alternating the time limits for individual presses. First, we changed the interval between the first and second press (L–L) to 1.5–5 s, forcing the mice to wait for an additional 1 s before the second press. The other intervals between presses remained unchanged (0.5–20 and 0.5–5 s, respectively).

##### LLRR RIGHT

This time, we changed only the last interval between the third and fourth press (R–R) to 1.5–5 s.

##### LLRR MIDDLE

Finally, we changed the interval between the two middle presses (L–R) to 3–20 s. After 3 d of testing, mice were moved to their last baseline sessions and then to the final stage.

##### Extinction

On the last day of our paradigm, we submitted mice to the extinction session with no reinforcement. The correct sequence was identical to the LLRR sequence with baseline time limits, including the cue lights and the 5 s TO after incorrect trials; however, no reward was delivered after completing a correct sequence.

### Timed sequence task: data analysis

The boxes were operated by the MedPC-V software that was also used to record all events that were defined in the task code. The resulting task log shows all successive events in a row (one row per session) as decimal numbers: the number before decimal point indicates the time of the respective event that elapsed from the last recorded event. The number after the decimal point indicates the specific event using a respective code as defined in the task. The task events recorded in the log included individual lever presses, both correct and incorrect, and presses during the TO interval, and reward delivery after the completion of correct sequences. Main summary parameters for each session (e.g., total number of presses) were recorded in a text file generated for each session. For more detailed analysis, we generated a comprehensive task log containing all sessions of the study and analyzed various performance parameters using the log and the analysis scripts created for that purpose. In addition to the individual analysis scripts, we also created a script allowing an easy and quick searching in the extensive task logs and visualization of defined time points during the session. The complete task log containing data from this study can be found at https://doi.org/10.5281/zenodo.7875357. A summary of all task codes and parameters we used is shown in Tables 2-Tables 4. The codes and the analysis scripts are available at https://doi.org/10.5281/zenodo.7881059.

**Table 2:** Task parameters used for the individual stages during the initial training

	Setting task parameters (first part)	
	Session time (min)	Maximum reinforcement
Habituation 1	10	
Habituation 2	10	
Habituation 3	30	15
Operant training	30	40

The depicted parameters follow the interface of the Med-PC V software (Med Associates). They may slightly differ when adapted to a different type of software.

**Table 3:** Basic task parameters used for the individual stages during the advanced phase of the training

	Setting task parameters (2nd part)									
	First correct lever (1 = left, 2 = right)	First correct lever (1 = left, 2 = right)	Reinforcement device (1 = pellet, 2 = dipper, 3 = drug)	Reinforcement time (s)	Time-out following reinforcement (s)	Session time (min)	First fixed ratio value	Second fixed ratio value	Maximum reinforcement	Softcr data array (1 = yes, 0 = no)
LR sequence										
LLR sequence										
LLRR sequence										
LLRR with baseline time limits										
LLRR with stricter time limits										
LLRR alternating: 3×LLRR + 1× LLR	1.000	0.000	1.000	0.050	0.000	60.00	2.0	1.0	40.000	1.000
LLRR *LEFT*										
LLRR Right										
LLRR *MIDDLE*										
Extinction										

The depicted parameters follow the interface of the Med-PC V software (Med Associates). they may slightly differ when adapted to a different type of software.

**Table 4:** Timing task parameters used in advanced stages of the training

	Tas parameters (third part)							
Setting	Time-out low left	Time-out high left	Time-out low right	Time-out high right	Time-out low 1st right	Time-out high 1st right	Time-out low 2nd right	Time-out high 2nd right
LR sequence								
LLR sequence	0.050 s	3600 s	0.050 s	3600 s				
LLRR sequence					0.050 s	3600 s	0.050 s	3600 s
LLRR with baseline time limits		5.000 s				20 s		5.000 s
LLRR with stricter time limits	0.500 s	2.000 s				10 s		2.000 s
LLRR alternating: 3× LLRR + 1× LLR					0.500 s		0.500 s	
LLRR *LEFT*	1.500 s							
LLRR *RIGHT*		5.000 s				20 s	1.500 s	5.000 s
LLRR *MIDDLE*	0.500 s				3.000 s		0.500 s	
Extinction					0.500 s			

The depicted parameters follow the interface of the Med-PC V software (MedAssociates). they may slightly differ when adapted to a different type of software.

### Statistical analysis

We used 22 mice in total, 11 mice per group (control vs VPA). Outliers were defined as values outside the range (average ± 2 × SD), and they were excluded from the analysis. The identified outliers are indicated in figure legends together with respective sample sizes. Statistical analysis was performed with GraphPad Prism version 8 (GraphPad Software). We used two *t* tests and one-way ANOVAs for evaluating the effect of one variable and two-way ANOVA or repeated-measures (RM) two-way ANOVA for evaluating the effects of two variables. Sidak’s test was used for *post hoc* comparison. For the analysis of the cumulative data, we used a nonparametric Mann–Whitney test to compare individual data points between individual groups of animals. Significance was set at *p* < 0.05, and the data in graphs are presented as the average ± SEM. Results of statistical analyses are reported as 95% confidence intervals (CIs) for the difference between means and respective *p* values.

### Data availability

The code used to run the task is available at https://doi.org/10.5281/zenodo.7881104. The scripts for data analysis are available at https://doi.org/10.5281/zenodo.7881059. The raw data presented in the article are available at https://doi.org/10.5281/zenodo.7875357.

## Results

### Evaluation of the autism-like phenotype in the VPA mice

**Figure 3.**
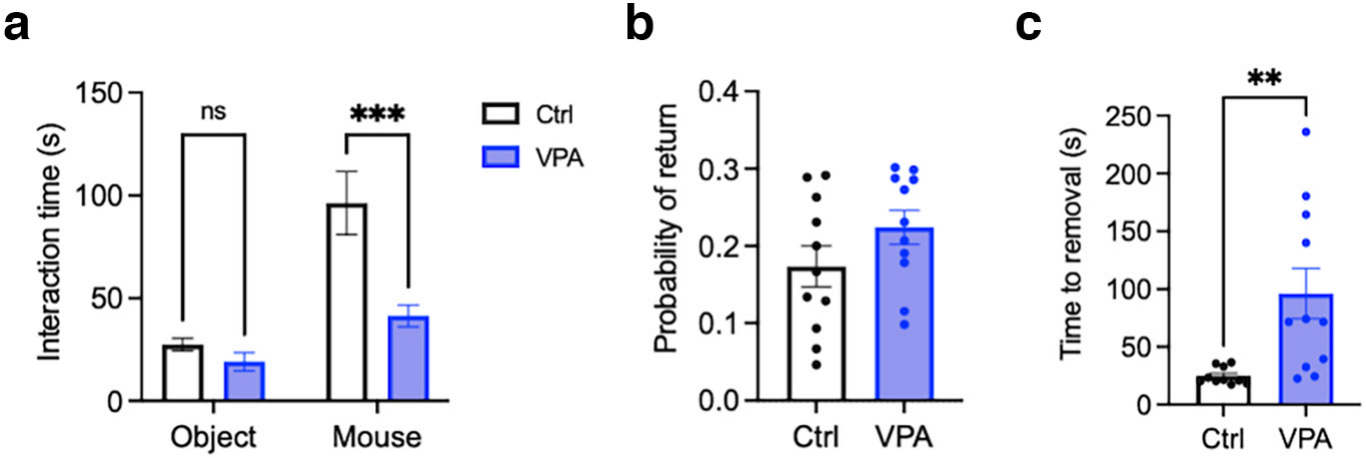
VPA mice show social impairment and reduced dexterity. ***a***, Interaction times during the social preference test in VPA and control mice. *n*_(VPA, ctrl)_ = 11. Effect of treatment: 95% CI, 14.4, 49.0; *p* = 0.0007; effect of stimulus: 95% CI, −62.9, −28.3; *p* < 0.0001; two-way ANOVA. ***b***, Probability of returning to the same hole during the hole board test in VPA and control mice. *n*_(ctrl, VPA)_ = 11. 95% CI, −0.02, 0.12; *p* = 0.157; two-tailed *t* test. ***c***, Time to remove tape in VPA and control mice during the tape removal task. *n*_(ctrl)_ = 10 (1 outlier excluded), *n*_(VPA)_ = 11. 95% CI, 22.9, 1119.7; *p* = 0.0061; two-tailed *t* test. All data are shown as the mean ± SEM. **p < 0.01; ***p < 0.001; ns ≥ 0.05. ctrl, Control.

First, we used the social preference test, hole-board test, and sticky tape test to confirm the mice prenatally exposed to VPA show the typical autism-like phenotype. Results indicating social and dexterity impairment are shown in Figure 3.

### Both control and VPA mice can learn the timed sequence task, and they improve during training

**Figure 4.**
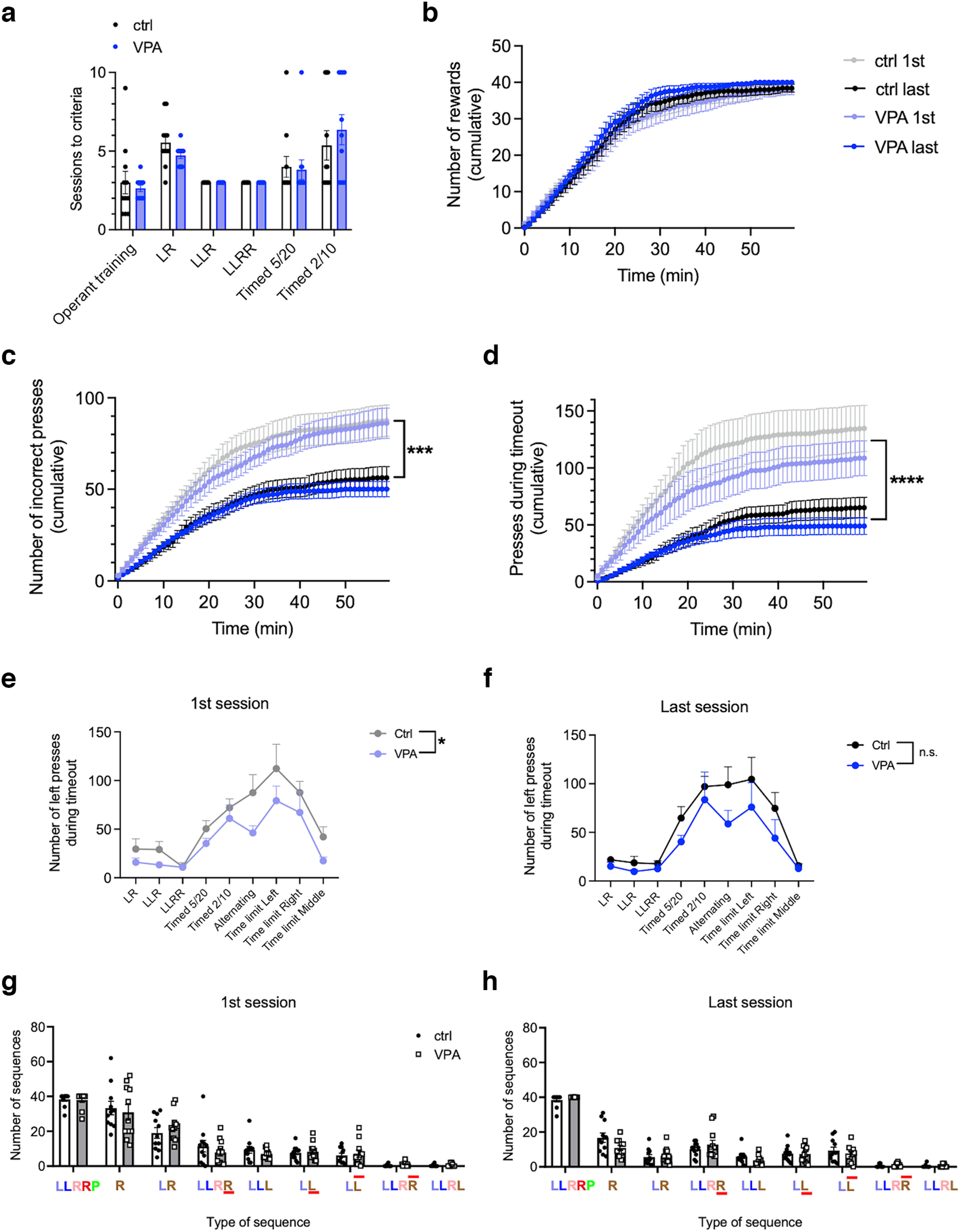
VPA and control mice learn successfully at all stages of the timed sequence task. ***a***, Number of training sessions needed to reach performance criteria in individual training stages. ***b–d***, Cumulative graphs showing the number of rewards earned (***b***), incorrect presses (***c***), and presses during time-out (***d***) emitted by VPA and control mice in the first session (light symbols) and last session (dark symbols) of the baseline timed LLRR sequence. ***e***, ***f***, Number of left time-out presses emitted on the first session (effect of treatment: 95% CI, 0.995, 38.0; *p* = 0.0399; ***e***) and last session (effect of treatment: 95% CI, −6.41, 42.2; *p* = 0.1403; ***f***) at different stages of training. ***g***, ***h***, Different types of pressing sequences performed on the first (***g***) and last (***h***) session of the baseline timed LLRR sequence. LLRRP, Correct sequence followed by a reward (“Pellet”); R, sequence started incorrectly with Right press; LR, second press incorrect Right; LLRR, fourth press too fast (below threshold); LLL, third press incorrect Left; LL, second press too fast (below threshold); L $\bar{L}$, second press too slow (above threshold); LLR $\bar{R}$, fourth press too slow (above threshold); LLRL, fourth press incorrect Left. In all graphs, *n*_(ctrl, VPA)_ = 11. Data are shown as the mean ± SEM, and they were analyzed by two-way ANOVA (***g***, ***h***), RM two-way ANOVA (***a***, ***e***, ***f***), or Mann–Whitney test (***b–d***). *p < 0.05; ***p < 0.001; ****p < 0.0001. ctrl, Control.

**Figure 5.**
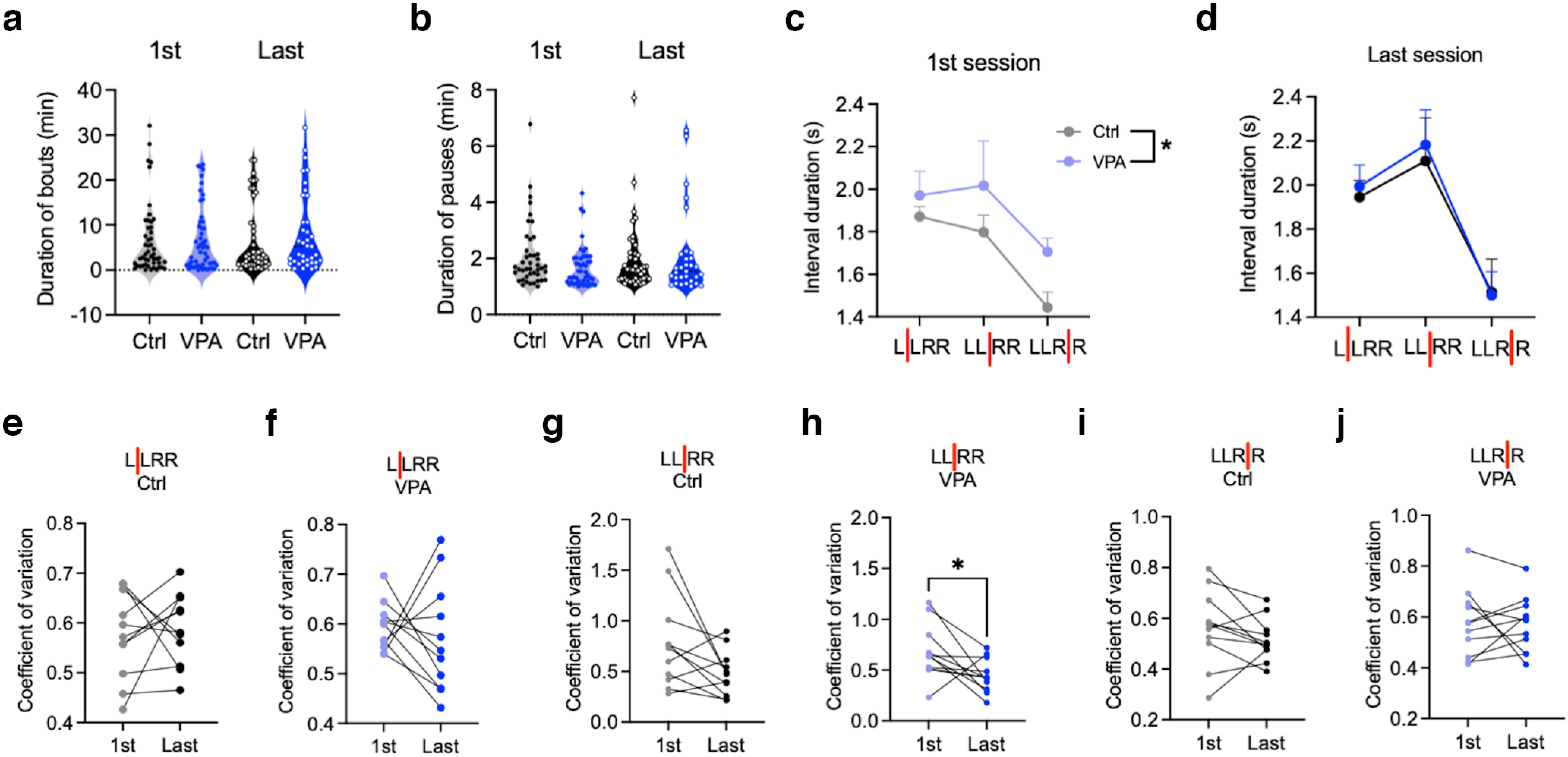
VPA mice show longer interpress intervals and decreased variability during specific stages of training. ***a***, ***b***, Duration of pressing bouts (***a***) and pauses (***b***) during the first and last session of the baseline timed LLRR sequence. ***c***, ***d***, Duration of individual interpress intervals during the first session (effect of treatment: 95% CI, −0.376, −0.011; *p* = 0.0381; effect of interval, *p* = 0.0037; ***c***) and last session (effect of treatment: 95% CI, −0.259, 0.187; *p* = 0.750; effect of interval, *p* < 0.0001; ***d***) of the baseline timed LLRR sequence. ***e–j***, Comparison of the variability of interpress intervals in VPA (***f***, ***h***, ***j***; in ***h***: 95% CI, −0.452, −0.014; *p* = 0.0391) and control (***e***, ***g***, ***i***) mice during the first and last session of the baseline timed LLRR sequence. In all graphs, *n*_(ctrl, VPA)_ = 11. Data are shown as the mean ± SEM, and they were analyzed by one-way (***a***, ***b***) or two-way (***c***, ***d***) ANOVA or by paired *t* test (***e–j***). *p < 0.05. ctrl, Control.

All mice met the habituation criteria and finished the subsequent training. The habituation was usually completed within one session, only in the VPA group, one mouse 1× repeated the Habituation 2 session, and 1× the Habituation 3 session. There was no significant difference between the control and VPA mice in the number of sessions needed to reach the training criteria (Fig. 4*a*). Both groups also significantly improved their performance during training. We compared their performance in the first and the last session of the “LLRR baseline” timed paradigm; that is, in the first timed training session and in the last baseline session, just before the extinction. While mice were reaching the same number of rewards in both sessions (Fig. 4*b*), both control and VPA mice significantly decreased the number of incorrect presses and presses emitted during the time-out (TO presses; Fig. 4*c*,*d*). Initially, VPA mice performed fewer TO presses compared with controls, but the difference was less prominent after training (Fig. 4*e*,*f*). To understand the activity of mice during the session, we quantified individual types of pressing sequences, correct and incorrect, performed in the first and last LLRR baseline session. Unlike the first session, in the last session mice performed significantly more correct sequences than any other type of sequence (Fig. 4*g*,*h*). During sessions, both groups often alternated periods of activity (bouts) and inactivity (pauses). Most of the bouts and pauses were relatively short (<5 and 2 min, respectively), and their duration did not differ between control and VPA mice (Fig. 5*a*,*b*). We also analyzed intervals between the individual presses in the first and last session of the LLRR baseline paradigm, only in correctly executed sequences. Although the required intervals between the two left and the two right presses were identical, both control and VPA mice were significantly faster to emit the second right press, especially after training (Fig. 5*c*,*d*). Initially VPA mice showed longer intervals between presses compared with controls but during training, the average interpress intervals in VPA and controls became indistinguishable (Fig. 5*c*,*d*). To assess the potential increase in stereotypical behavior during training, we checked the variation coefficients of individual interpress intervals during the first and the last session of the LLRR baseline paradigm, and we found a decrease in the variability in the middle interval (L–R) in VPA animals only (Fig. 5*e–j*).

### VPA mice show better adaptation to changed pressing times but not changed pressing sequence

**Figure 6.**
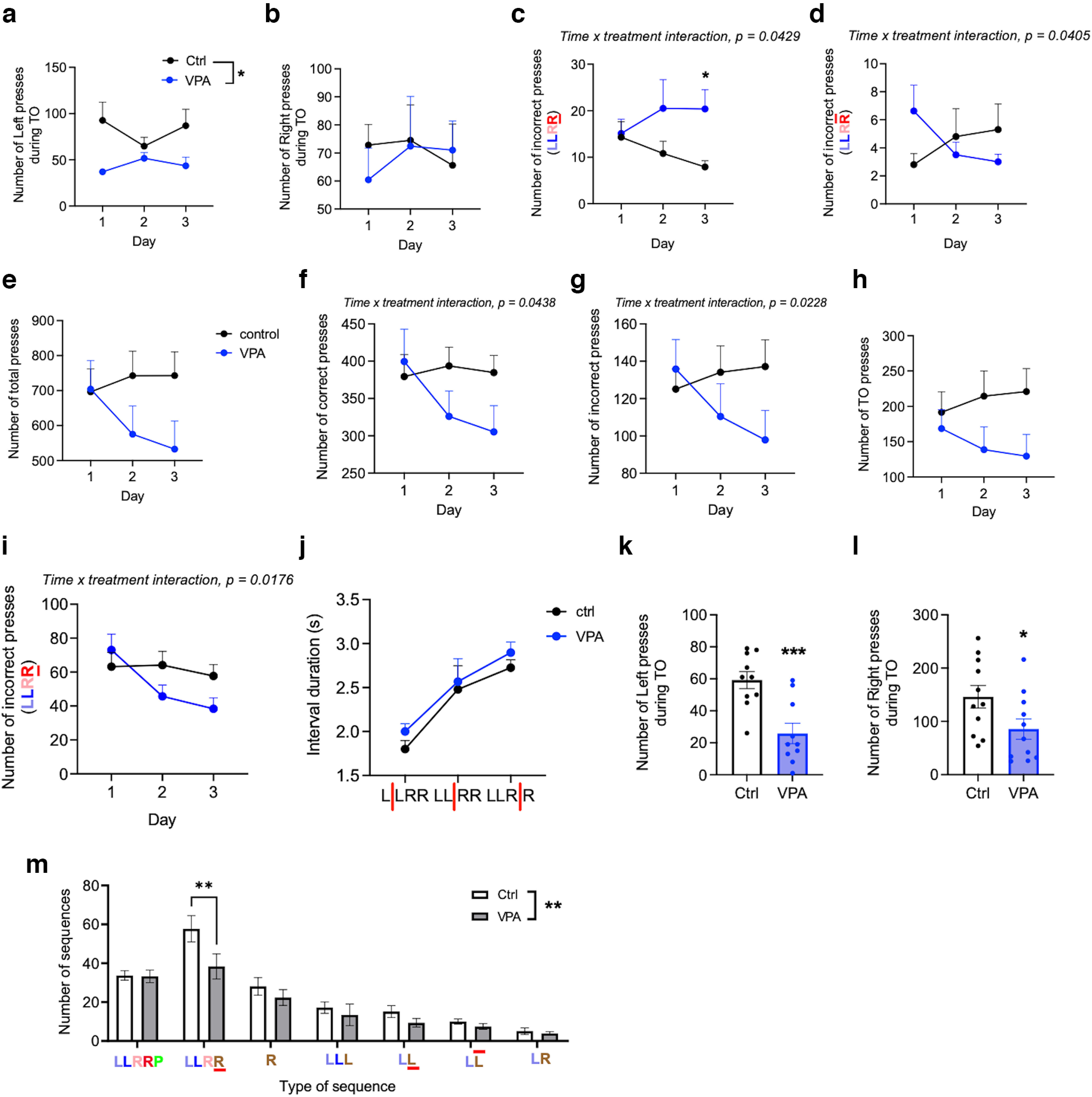
VPA mice show better adaptation to the change of timing but not when the pressing sequence itself is changed. ***a–d***, Performance criteria during the LLRR alternating sessions: number of left presses (effect of treatment: 95% CI, 4.69, 70.10; *p* = 0.027; ***a***) and right presses (***b***) during time-out, and the number of fourth presses emitted too fast (***c***) and too slow (***d***). *n*_(ctrl)_ = 10 (1 outlier excluded from ***a*** to ***d***), *n*_(VPA)_ = 8–10 (2 outliers excluded from ***a*** to ***b***, 1 excluded from ***c***, and 3 excluded from ***d***). ***e–i***, Performance criteria during the three LLRR *RIGHT* sessions: number of total (***e***), correct (***f***), incorrect (***g***), and time-out (***h***) presses in each session; number of the fourth presses emitted too fast in each session (***i***). *n*_(ctrl, VPA)_ = 11. ***j–q***, Performance criteria during the last LLRR *RIGHT* session only. ***j***, Duration of individual interpress intervals. ***k***, ***l***, Number of left time-out presses (95% CI, −50.9, −15.9; *p* = 0.0008; ***k***) and right time-out presses (95% CI, −120.1, −1.163; *p* = 0.046; ***l***). ***m***, Different types of pressing sequences emitted (effect of treatment: 95% CI, 1.52, 9.57; *p* = 0.007). Sequence abbreviations are as in Figure 4. *n*_(ctrl)_ = 10–11 (1 outlier excluded from ***k***); *n*_(VPA)_ = 10-11 (1 outlier excluded from ***k***). Data are shown as the mean ± SEM, and they were analyzed by RM two-way ANOVA (***a–j***), two-tailed *t* test (***k***, ***l***), or two-way ANOVA (***m***). *p < 0.05; **p < 0.01; ***p < 0.001. ctrl, Control.

**Figure 7.**
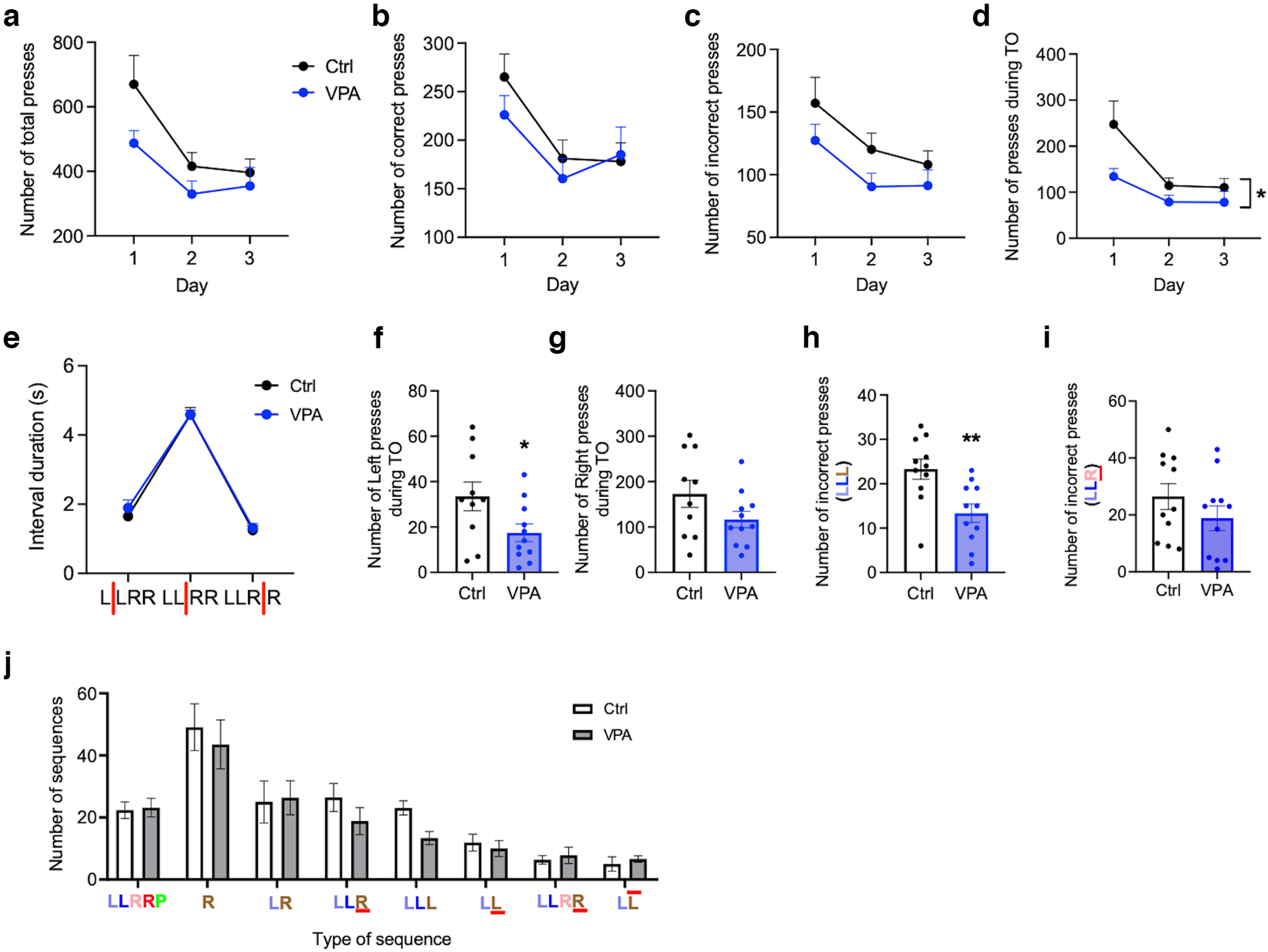
VPA mice maintain the original sequence when timing of the presses is changed. ***a–d***, Performance criteria during the three LLRR *MIDDLE* sessions. Number of total (***a***), correct (***b***), incorrect (***c***), and time-out (effect of treatment: 95% CI, 8.0, 112.9; *p* = 0.026; ***d***) presses in each session. ***e–n***, Performance criteria during the first LLRR *MIDDLE* session only. ***e***, Duration of individual interpress intervals. ***f***, ***g***, Number of left time-out presses (95% CI, −31.30, −0.79; *p* = 0.040; ***f***) and right time-out presses (95% CI, −127.7, 15.0; *p* = 0.115; ***g***). ***h***, Number of third presses incorrect Left (95% CI, −16.36, −3.46; *p* = 0.004). ***i***, Number of third presses emitted too fast (95% CI, −20.69, 5.42; *p* = 0.237). ***j***, Different types of pressing sequences emitted during the first LLRR *MIDDLE* session. Sequence abbreviations are as in Figure 4. *n*_(ctrl)_ = 10–11 (1 outlier excluded from ***f***, ***g***); *n*_(VPA)_ = 11. Data are shown as the mean ± SEM, and they were analyzed by RM two-way ANOVA (***a–d***), two-way ANOVA (***e***, ***j***), or two-tailed *t* test (***f–i***). *p < 0.05; **p < 0.01. Ctrl, Control.

After the mice showed reliable performance in the LLRR baseline paradigm, we manipulated either the sequence structure or the required interpress intervals. First, we implemented the LLRR alternating paradigm, where in every fourth trial the LLRR sequence was shortened to LLR (Fig. 6*a–d*). The occasional shortening of the pressing sequence affected control and VPA mice differently. During the 3 d of the LLRR alternating paradigm, controls decreased the number of premature presses and increased the number of delayed second right presses, while VPA mice did the opposite (Fig. 6*c*,*d*). After alternating the sequence, we changed the individual interpress intervals, specifically by prolonging the lower time limit and forcing animals to wait longer before the next press. First, we increased the lower time limit between the two left presses to 1.5 s, which had only modest effect on the performance of mice (data not shown). Next, we prolonged the lower time limit between the two right presses to 1.5 s. This affected performance of mice (Fig. 6*e–m*), but unexpectedly, VPA mice adapted better to this change and during the 3 training days, they decreased the number of incorrect presses (Fig. 6g,*i*). In particular, VPA mice were less prone to make a premature second right press (Fig. 6*i*,*m*). Finally, we changed the interval between the left and right middle presses by increasing the lower limit to 3 s, which proved to be the most challenging modification. Although VPA mice again adapted more easily to this change, the difference between groups was not so prominent (Fig. 7*a–j*). VPA mice did not make less premature first right presses compared with controls (Fig. 7*i*), but interestingly, they were less likely to change the sequence structure by adding a third left press (Fig. 7*h*). Overall, our data suggest that VPA mice can easily adapt to changes in the interpress intervals, but they are less likely to change the number and order of presses in the originally learned sequence.

### VPA mice are less affected by the omission of reinforcement in the extinction session

**Figure 8.**
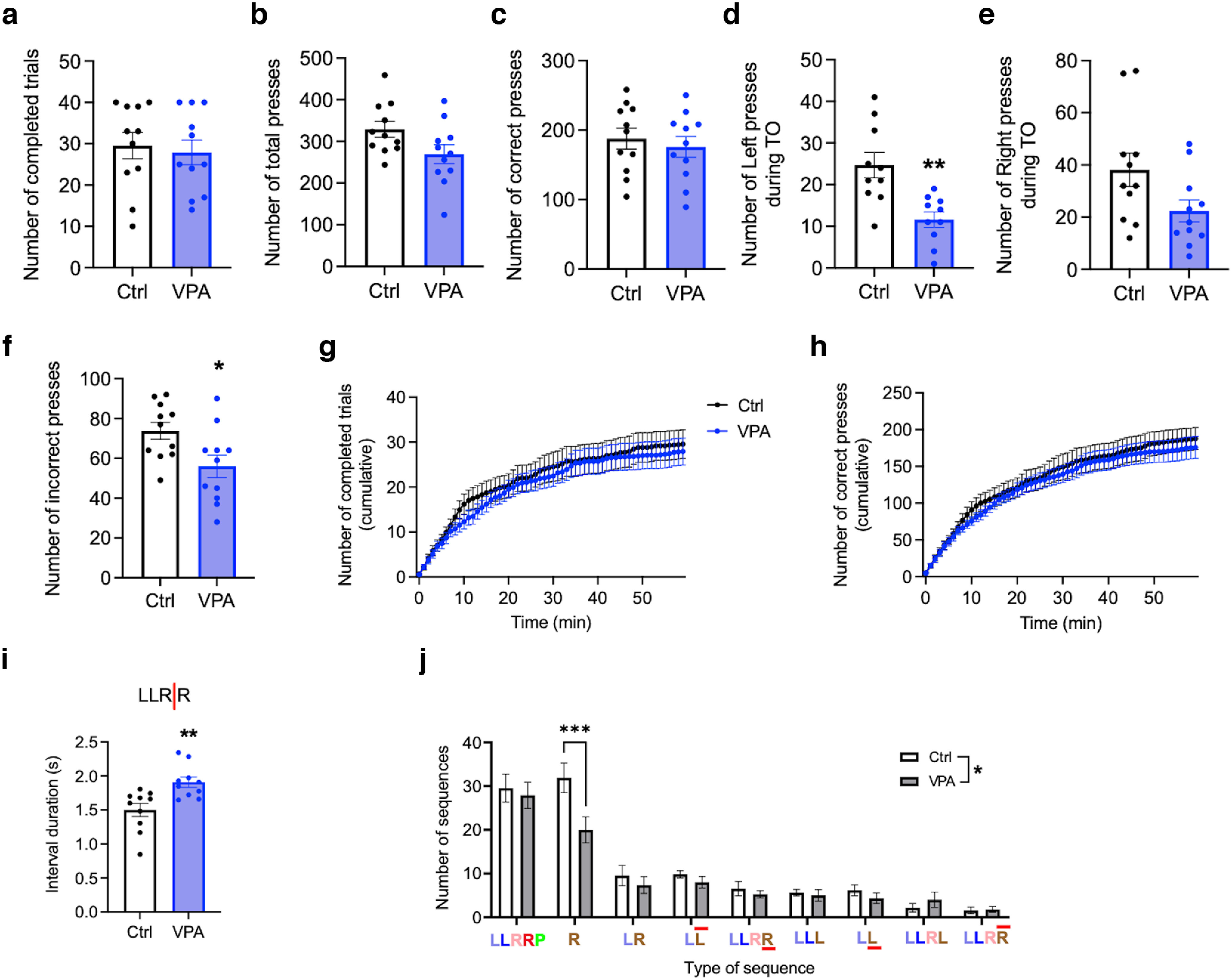
VPA mice maintain the original sequence when the reward is omitted during extinction session. ***a–f***, Performance parameters during extinction session. Number of completed trials (95% CI, −10.75, 7.47; *p* = 0.712; ***a***), total presses (95% CI, −120.6, 2.10; *p* = 0.058; ***b***), correct presses (95% CI, −56.5, 32.3; *p* = 0.576; ***c***), left presses (95% CI, −20.57, −5.63; *p* = 0.002; ***d***), and right presses (95% CI, −31.83, 0.195; *p* = 0.053; ***e***) during time-out and incorrect presses (95% CI, −32.6, −3.1; *p* = 0.021; ***f***). ***g***, ***h***, Cumulative graphs showing the number of completed trials (***g***) and correct presses (***h***). ***i***, Duration of the last interpress interval (95% CI, 0.151, 0.667; *p* = 0.0038. ***j***, Different types of pressing sequences emitted during the Extinction session. Effect of treatment (95% CI, 0.368, 3.895; *p* = 0.018). Sequence abbreviations are as in Figure 4. *n*_(ctrl)_ = 10–11 (1 outlier excluded in ***d***, ***i***); *n*_(VPA)_ = 10–11 (1 outlier excluded in ***d***, ***i***). Data are shown as the mean ± SEM, and they were analyzed by two-tailed *t* test (***a–f***, ***i***), Mann–Whitney test (***g***, ***h***), or two-way ANOVA (***j***). *p < 0.05; **p < 0.01; ***p < 0.001. ctrl, Control.

Finally, we performed a single extinction session to test whether VPA mice were more likely to maintain their performance in the absence of reward, which would be a sign of cognitive inflexibility (Mar et al., 2013). Control and VPA mice on average completed the same number of trials, and a similar proportion of mice in both groups did not complete the maximum 40 trials (Fig. 8*a*,*g*). In line with that, the number of total and correct presses in both groups also did not differ (Fig. 8*b*,*c*,*h*). However, VPA mice emitted fewer incorrect and TO presses (Fig. 8*d–f*). They were less prone to start a new sequence with the right press, after the reward failed to be delivered at the end of the sequence (Fig. 8*j*). Instead, VPA mice responded by prolongation of the last interpress interval (Fig. 8*i*), from ∼1.5 s (Fig. 5*d*) during the baseline performance to almost 2 s (Fig. 8*i*). In summary, similar to the previous sessions, VPA mice tended to maintain the original order of presses in the sequence while having less difficulty in changing the time intervals between them.

## Discussion

We present a new paradigm that allows the evaluation of motor learning and flexibility. The design of the task was motivated by the need to test a complex behavior in an automated and reproducible manner, while using commonly available equipment. To understand behavioral changes in various mouse models, it is necessary to evaluate multiple parameters. However, in the operant tasks, often only a few primary parameters such as accuracy are commonly analyzed, which may lead to the disregard of more subtle differences in behavioral strategies. For instance, we showed that in the VPA mice, the total number of earned rewards was indistinguishable from controls at all stages of the training. Upon closer look, however, the performance of the two groups was quite different, suggesting slower motor performance and decreased ability to adapt the originally learned sequence. Another significant difference between the VPA and control mice was the increased number of TO presses present at multiple stages of the training (Fig. 4*e*).

In our task, we take advantage of gradually increasing difficulty at different stages to reveal potentially gross or more subtle behavioral impairment, and we analyze multiple parameters to better understand its character. During the training, mice have to use basic operant learning, motor learning, time perception, habit formation, behavioral flexibility, and motivation. Importantly, even with only two fixed levers, the task consists of reward-distal and reward-proximal elements, and allows them to be analyzed separately (Keeler et al., 2014). Although the two left and the two right presses were seemingly symmetrical and the required pressing intervals between them were identical at baseline, mice were clearly able to distinguish that their distinct reward proximity as the average interpress interval between the two right presses was significantly shorter (Fig. 5*d*). To use the timed sequence task for the evaluation of flexibility, we need to make sure the performance of mice is becoming automated and habitual during the training. To test this, we compared the variability of individual pressing intervals in the first and the last LLRR baseline session (Fig. 5*e–j*). Surprisingly, we did not find any decrease of variability in control animals, possibly because the pressing intervals were largely constricted by the predefined criteria and there was little space for further improvement. In addition to the low variability of individual motor actions, the automated character of behavior is indicated by an increase in speed and accuracy (Turner et al., 2022). In our experiment, we observed a significant increase in accuracy (Fig. 4*c*) and efficiency, as mice performed markedly fewer TO presses in later stages of the training (Fig. 4*d*).

There are currently several alternatives that allow a detailed testing of motor or operant learning and flexibility. Among those, the touchscreen-based systems offer a great number of predesigned paradigms and allows creating additional, custom-made tasks (Horner et al., 2013; Mar et al., 2013; Heath et al., 2016; Janickova et al., 2021). However, a significant cost of such systems may be prohibitive for some users. Standard operant boxes, on the other hand, are easily accessible to most laboratories. We show here that even the most basic operant box equipped with two fixed levers and one feeder can be used to perform a relatively complex task and collect detailed behavioral data. We provide a computer code for the task itself and also a set of scripts for data analysis, allowing easy and fast processing of large datasets. In the mice we tested, completion of the whole paradigm took ∼45 training days. This is more than in standard operant learning tasks but comparable to other more sophisticated paradigms, offering detailed insight into the behavior of mice. One of the advantages of our paradigm is that it is testing the performance of the animals over the course of several weeks at different levels of difficulty, and it records and analyzes multiple behavioral parameters. It makes it perfectly suited to detect more subtle changes in behavior, compared with one-session tasks such as the hole-board test. Because of its long-term character, it can also determine whether specific behavioral changes can manifest temporarily at the beginning of training or whether they are more consistent, suggesting a permanent cognitive trait.

A potential limitation of the study is our focus on examining only males. We decided to validate our paradigm in males because of the higher prevalence of autism spectrum disorder (ASD) in men (Werling and Geschwind, 2013). In addition, most studies did not find a difference in the behavioral phenotype between sexes after prenatal VPA exposure (Ergaz et al., 2016). However, conducting the paradigm on females would be intriguing since they can exhibit lower cognitive flexibility than males (Korol et al., 2004; LaClair et al., 2019). In addition to sex, another variable to consider is the influence of circadian rhythm. Mice typically exhibit higher activity during nocturnal periods. Additionally, VPA-treated C57BL/6 mice displayed decreased place relearning and an increased tendency to perseveration, particularly during light periods (Puścian et al., 2014). These findings align with observations that people with ASD often display nocturnal activity and, during the day, they have increased melatonin levels, which contribute to emotional or cognitive problems (Carmassi et al., 2019).

In summary, we provide a new, easily applicable paradigm for detailed testing of motor learning and flexibility. We provide tools for data analysis, arguing that multiple parameters should be used to evaluate the performance the animals in a complex task. Finally, we provide an example showing that our paradigm can reveal subtle differences in mouse performance and help to identify general behavioral mechanisms underlying those differences.

## Synthesis

Reviewing Editor: Nathalie Ginovart, Universite de Geneve Decisions are customarily a result of the Reviewing Editor and the peer reviewers coming together and discussing their recommendations until a consensus is reached. When revisions are invited, a fact-based synthesis statement explaining their decision and outlining what is needed to prepare a revision will be listed below. The following reviewer(s) agreed to reveal their identity: Sreemathi Logan.

The reviewer expressed interest in your manuscript but also raised a number of issues that should be addressed in a revision. Importantly, the methods are not abundantly clear and lack important information, which undermines the impact of the work. The statistics should also be revised. Moreover, although the visual abstract is informative in conveying the experimental paradigm, it would be more informative to add readouts of the design (cognitive flexibility) and applicability of the readouts.

Please find the specific comments and queries raised by the reviewers below:

Reviewer 1:

The authors designed a new behavioral testing paradigm for operant conditioning with a timed sequence for motor learning to discriminate subtle behavioral changes in cognitive flexibility. They use young (∼2 mo) C57Bl/6J mice and a model of autism (VPA induced) and show differences in time differences for sequence performance between VPA and control mice that may otherwise be overlooked in standard testing. This testing paradigm provides a valuable tool that can be adapted to standard operant behavioral testing apparatus.

Many aspects of the methodology needs to be clarified.

Methods section:

Were the mice testing during daytime hours? Please indicate hours of the day that testing was performed.

Please indicate how many mice of the total ate all pellets in Habituation 2 and advanced to the next stage. How many times was Habituation 2 repeated and was it repeated until all mice ate their pellets? How many mice received repeated habituation vs. how many did not receive repeated habituation.

How many mice of the total advanced from Habituation 3? Was habituation 3 repeated for those that did not complete the task?

Please describe how the 40 pellet criteria was determined.

LLR sequence: is the 40 rewards per day for 3 consecutive days? Or a total of 40 rewards over the 3 days? Please clarify this for other sequences as well.

Please revise sentence “During the TO interval, potential lever presses were recorded but they had no consequences”, as lack of a reward is a negative consequence. Is this not similar to extinction where no rewards are provided.

Other tests run on VPA mice: Please describe social preference, hole board and sticky tape tests as they were performed for these mice. While references are provided, please indicate specifically how these tests were performed for this study design, age of mouse, sequence of tests, etc.

Statistical analyses:

Based on the study design, a repeated measures ANOVA should have been used. Also please clarify as to how the stats were done on the cumulative data.

Figures

Figure 7 subparts are not referenced in the text. Only Figure 7 i and h are referenced. Some Figure 8 subparts are not referenced.

Discussion

Please indicate the total number of days for the entire testing paradigm and discuss the feasibility of such a long and complicated testing paradigm.

Please discuss day/night testing and how that can affect results.

Please discuss sex-specific cognitive flexibility and sensitivity using the current paradigm in females.

Since the VPA model of autism used in this study already shows neurocognitive deficits (using other tests), please discuss the validity of detecting subtle changes in cognitive flexibility in this model and the application of this test as an early detection and predictability of future neurocognitive deficits.
